# Eczema: Its Local and General Treatment

**Published:** 1907-08-17

**Authors:** Morgan Dockrell

**Affiliations:** Physician to St. John's Hospital for Skin Diseases, Chesterfield Lecturer on Diseases of the Skin


### HOSPITAL CLINICS.

### ECZEMA: ITS LOCAL AND GENERAL TREATMENT.

By MORGAN DOCKRELL, M.A., M.D.(Dub.), Physician to St. John's Hospital for Skin Diseases, Chesterfield Lecturer on Diseases of the Skin.

### General Treatment.

Dr. Dockrell commenced by stating that in the treatment of eczema attention to detail was of the utmost importance. There was nothing so slight or so trivial that it could with safety be neglected.

*Washing.*—It is important that the patients should not wash themselves too frequently. On the other hand, there is now a tendency to advise them not to wash at all, and this is a far greater mistake than to let them wash too frequently. The commonest question so far as washing in water is concerned, is whether the patient should take an ordinary bath every morning. This may be allowed if a little ointment is applied to the parts before the patient goes into the bath. The part is thus protected and the delightful sensation and moral effect of the bath are obtained.

*Soap.*—This is a very important agent. Use soaps that have an excess of fat. Civilisation has made our skins rather tender. It is important that they should be kept more or less anointed, and for this purpose a super-fatted soap should be used. Purified coal-tar—a delightful application as a rule—is rather a worry and anxiety when used in the form of soap, and may be responsible for keeping up eczematous trouble.

Rest plays a very important part in the treatment of eczema. A large number of cases will not get well except by soothing baths and rest in bed. Wearing apparel is also important. Patients who suffer from chronic eczema or have thin skins are unable to wear wool next their skin. Locality and residence are of some importance. Bracing air, for example that of the Downs, is better for eczematous people than sea air.

Water internally plays an important part in the general health of those affected with eczema, and thus it is advisable to ascertain whether patients are possibly affected by the water supply of the place they live in. Hard water, used for ablutions or for culinary purposes, tends to cause some people to suffer from eczema.

Digestive conditions affect the general health and must be carefully looked to, as an improper diet frequently lights up eczema. When patients suffer from eczema they are better without uncooked fruits and vegetables; they should also avoid all salt foods and, I think, in a large number of cases tea and coffee. As to beverages, weak claret and an occasional glass of port are often useful in eczema, though generally speaking the trend of opinion is that these patients are better without stimulants.

*Medicinal (Internal) Treatment.*—There is no specific for eczema. It must be treated on general lines and chiefly locally. Very few internal remedies are prescribed, but in acute conditions vinum antimoniale with sulphate of magnesium mixture may be administered for the purpose of depressing the circulation and thus cutting short the attack. A considerable amount of itching is usually present, and as the above drugs are useful in diminishing arterial tension, they help to relieve the itching. The tincture of gelsemium also relieves itching, and in some cases five minims of the tincture of nux vomica does the same. Certain drugs, notably arsenic, are supposed to have a specific effect. Arsenic is always contra-indicated in active proliferation, and indeed has very little scope, except in the eczema of children, when Fowler's solution is useful. If nervous energy seems to be below par, Fellows' syrup may be used. Where the skin shows marked deficiency of secretion, a sixth of a grain of pilocarpine may be given.

Eczema of children has been divided by Unna into three varieties: (1) nervous, (2) tuberculous, (3) seborrhœic. Nervous eczema is associated with teething. It occurs usually on the cheeks, on the radial side of the wrist, and on the backs of the hands. In these cases Fowler's solution in two minim doses is very useful. It may be associated with two grains of bromide of potassium in two teaspoonsful of water for a child one year old, and administered three times a day. Locally, an ointment consisting of vaseline with 1 per cent. of resorcin is to be applied.

Tuberculous eczema occurs around the mouth in children who are apparently well nourished, but who are mostly brought up on milk and jam. It shows a marked tendency to persist. It improves when the child is sent to the seaside. Ichthyol internally, five grains, is useful in such a case. Locally you will find resorcin of service.

The so-called seborrhœic eczema generally occurs over the fontanelles and spreads to other parts; the fatty acids in the seborrhœic secretion set up irritation of an eczematous character and having a tendency to extend. Arsenic is of great value in such cases, and resorcin ointment (1 per cent.) should be used locally. This ointment is antiseptic and tends to thicken the horny layer of the skin, and thus helps to resist the local irritation. In young children, eczema is very commonly complicated with pustules due to a secondary inoculation. These should be treated with mercurial ointment. There is no such thing as exclusively pustular eczema.

Climacteric eczema is supposed to occur in women about the age of forty or fifty. I do not think there is much in this form of eczema. It occurs in women and affects the legs; if treated with Fowler's solution and vinum ferri matters rapidly improve. Apart from eczema in children, this is the only occasion where it seems necessary to use arsenic; in other conditions it does harm.

### Local Treatment.

A few hints may be given as to local treatment. Be careful to ascertain the peculiarities of the patient's skin. If you take a little trouble about this you are soon able to sum up the character of a patient's skin, and to ascertain whether it is thin or thick. If you stimulate a thin skin conditions are made worse, and if you soothe a thick skin no advantage is gained.

In acute or sub-acute varieties of eczema the best applications to use are dusting-bags. Dusting-bags are simply bags filled with starch; they are applied to the surface, as in the axilla, and are most useful in the erythematous variety of eczema. The following are the commonest powders: starch, powdered talc, oleate of zinc, carbonate of zinc, and Taylor's similac powder. A useful formula is:—

| | | |
|---|---|---|
| Bismuth subnitrate ... ... ... | drs. | 2 |
| Powdered starch... ... ... ... | ,, | 7 |
| Acetate morphia ... ... ... ... | gr. | 1 |

Another is:—

| | | |
|---|---|---|
| Camphor ... ... ... ... ... | dr. | ½ |
| Rectified spirit q.s. | | |
| Powdered talc and zinc oxide, of each | drs. | 6 |

Always remove the dusting powder from the skin once in twenty-four hours.

*Lotions.*—In the application of lotions, free evaporation should be allowed; therefore only a very thin gauze is suitable for applying a lotion. Lotions should never be applied by soaking lint in them and then applying the soaked lint to the skin. The only time this is at all permissible is in the removal of crusts, when boracic-acid lotion is used. Instead of applying on gauze the lotion may be brushed on the skin and then allowed to dry. To soothe and cool the skin, thin, warm, rice water should be used. Boric acid (2 drams to the pint) is very comforting if sponged over the surface and afterwards dried off. Bicarbonate of sodium, hyposulphite of sodium (of each 2 drams to the pint), are also useful if sponged over the surface and the surface dried. Carbolic acid (1 dram to the pint of water) is beneficial.

In vesicular and papular varieties of eczema the following may be used:—

| | | |
|---|---|---|
| Liquor carbonis detergens ... ... | dr. | ½ |
| Glycerine ... ... ... ... ... | oz. | ½ |
| Water ... ... ... ... | to oz. | 6 |

This should be dabbed over the surface. If it is found unduly irritating, suspect the glycerine. Another useful lotion is—

Liniment of lime and black wash, of each oz. 4
Mucilage of tragacanth 1 dr. should be added.

One of the most useful lotions is the lotio calaminæ co.:—

| | | |
|---|---|---|
| Carbonate of zinc ... ... ... | drs. | 4 |
| Oxide of zinc ... ... ... ... | ,, | 2 |
| Glycerine ... ... ... ... ... | ,, | 1½ |
| Rose-water ... ... ... | ...to oz. | 6 |

This should be well shaken up and applied with a brush. It is useful not only as a protective to the skin, but tends also to make the skin look uniform and enhances its beauty. If there is much itching you may add the liquor carbonis detergens 30 minims.

*Ointments in Acute and Sub-acute Eczema.*—These should never in skin diseases be applied on lint or linen; butter-muslin is the best. In erythematous eczema, cold cream is an excellent basis for soothing ointments:—

| | | | |
|---|---|---|---|
| Spermaceti and white wax ... | of each | dr. | 1 |
| Almond oil and rose-water ... | ,, | ,, drs. | 10 |

In applying ointments do not use an excessive quantity; the skin will only take up a certain amount of grease at a time.

In eczema of the axilla or the groin, or of parts where two surfaces are in close contact, the following ointment is useful:—

Lead plaster and vaseline equal parts.

If something very soothing is required, the following is a suitable preparation:—

| | | |
|---|---|---|
| Powdered camphor ... ... ... | grs. | 30 |
| Oxide of zinc ... ... ... ... | drs. | 2 |
| Glycerine ... ... ... ... | ,, | 2 |
| Benzoated lard ... ... ... ... | oz. | 1 |
| Cochineal ... ... ... ... | gr. | 1 |
| Oil of roses ... ... ... ... | min. | 1 |

In papular eczema an ointment largely used is—

| | | |
|---|---|---|
| Ammoniated mercury ... ... ... | grs. | 5 |
| Liquor carbonis detergens ... ... | dr. | ½ |
| Vaseline ... ... ... ... | ...to oz. | 1 |

This is a very useful formula to remember, because it can be applied in many cases.

In some diseases it may be necessary to add chrysophanic acid; and it makes an excellent ointment for getting rid of infiltration if salicylic acid is added to it. In other cases it may be necessary to omit the liquor carbonis detergens.

Another good formula for papular eczema is:—

| | | |
|---|---|---|
| Liq. carbonis deterg. ... ... ... | dr. | 1 |
| Zinci oxid. ... ... ... ... | ,, | 1 |
| Ung. rosæ ... ... ... ... | oz. | 1 |

Hydro-naphthol (2 per cent. in vaseline) is applicable where there is much itching.

*Vesicular Eczema.*—Olive oil and lime-water in equal parts are recommended by many. Carbonate of lead and carbonate of zinc (2 drachms of each with 4 ounces of olive oil) are also advised. The ointment of ichthyol (30 per cent. in vaseline, with or without 1 dram of oxide of zinc added) is an application that does much good in many cases in vesicular eczema, especially if the patient's skin is delicate—if otherwise ichthyol may be used without the zinc oxide.

*Chronic Forms of Eczema.*—In chronic eczema, baths require consideration. These may be vapour, plain, or medicated. Perhaps everything required is found in the plain vapour bath. You will find that a hot-air bath in some cases is very useful. The ordinary box-bath is very useful if used until the perspiration becomes free. A radiant-heat bath is also very important in chronic eczema, but many people cannot stand this, and one has to fall back on the hot-air or vapour bath. Baths remove crusts, allay irritation, stimulate the lymphatics, and increase activity of the skin. An important point about the radiant-heat bath is that it should be followed by the hot-water bath. The weak part of the radiant-heat treatment is that the subsequent washing is omitted, and the acid perspiration irritates the skin.

You are sometimes recommended to use certain medicated soaps—ichthyol, salicylic acid, and resorcin soaps. Personally I think this is a great mistake. For infiltrated patches the following has been advised:—

| | |
|---|---|
| Oil of cade ... ... ... ... ... | dr. 1 |
| Verbena ... ... ... ... ... ... | min. 5 |
| Benzoated lard ... ... ... ... ... | oz. 1 |

In irritable and infiltrated conditions of the skin naphthol has been highly recommended.

*Localised Eczema.*—Eczema of the ear involving the auricle, if acute, requires soothing applications. If chronic, the diachylon ointment and vaseline with resorcin (3 per cent.) is useful. If the disease involves the meatus, brushing with glycerine and tannic acid is beneficial.

For eczema of the nostrils, glycerine and tannic acid is useful. If a chronic catarrhal condition of the mucous membrance is present the nose should be syringed out with a solution of boric acid, one dram to the ounce of water. Neuymann recommends a nasal suppository made of 2 grains of oxide of zinc and cacao-butter. Eczema of the lips is troublesome, specially amongst motorists. You will find that the tincture of benzoin is useful to paint on the surface, and the fluid extract of geranium painted into the fissures also does good.

Eczema of the breast and nipples usually occurs during the suckling period. It may be treated with a nitrate of silver pencil or by the application of compound tincture of benzoin, or of glycerine of tannic acid. The following formula is also recommended as a paint:—

| | |
|---|---|
| Acid. tannic. ... ... ... ... | grs. 20 |
| Ext. bellad. ... ... ... ... | ,, 10 |
| Ext. opium ... ... ... ... | ,, 5 |
| Aqua rosæ ... ... ... | ad ,, ℨss. |

Eczema near the anus can be treated by promoting regular movement of the bowel by means of saline tartrates and sulphates. Various ointments have been recommended.

| | |
|---|---|
| Cocaine ... ... ... ... ... ... | dr. 1 |
| Olive oil ... ... ... ... ... ... | drs. 2 |
| Lanoline ... ... ... ... ... | to oz. 1 (qy. 10) |

In eczema of the genitals cleanliness and the use of 1 or 2 per cent. resorcin ointment may be tried. In eczema of the vulva always examine for sugar. Painting with a nitrate of silver solution (16 grains to the ounce of spirit of nitrous ether) is of great benefit.

In eczema of the trunk the diachylon ointment is employed. In local bleeding patches the German School recommends a paste made as follows:—

| | |
|---|---|
| Zinc oxide ... ... ... ... ... | drs. 3 |
| Salicylic acid ... ... ... ... ... | ,, 11 |
| Benzoated lard ... ... ... ... ... | oz. 3½ |

For squamous patches the following is recommended:—

Precip. carbonate of calcium, oxide of zinc, oil of linseed, and lime water ... ... ... of each dr. 1

Lassar's paste is used where the patches are dry:—

| | |
|---|---|
| Salicylic acid ... ... ... ... ... | grs. 10 |
| Vaseline ... ... ... ... ... ... | oz. ½ |
| Oxide of zinc ... ... ... ... ... | drs. 11 |
| Powdered starch ... ... ... ... | ,, 11 |

The following is a very convenient medium for the application of certain remedies:—

| | |
|---|---|
| Gelatine ... ... ... ... ... ... | drs. 4 |
| Zinc oxide ... ... ... ... ... | ,, 3 |
| Glycerine... ... ... ... ... ... | ,, 5 |
| Water ... ... ... ... ... ... | ,, 9 |

Should you desire to use precipitated sulphur or ichthyol with this, the formula should contain one part less of gelatine.

In papular eczema employ:—

| | |
|---|---|
| Liq. carb. deterg. ... ... ... | oz. ½ |
| Liq. plumbi subacetatis ... ... | ,, 2½ |

One teaspoonful in a pint of warm water.

This should be sponged over the limb and the skin then dried, and some soothing application applied. In papular eczema you will find the gelatine of ichthyol useful, and also the gelatine of hydronaphthol.

In eczema rubrum, in which you have a complete denudation of the epidermis, you will find cold starch poultices very useful. The great object is gradually to get the epithelium to grow over the surface, and then to strengthen it by other applications. The following is the formula for making a cold starch poultice:—

One teaspoonful of the best white starch heaped up; mix this with a little water, fill up to a pint with boiling water, and let it stand until it gets cold.

Spread on butter-muslin and apply it. It is useful in all cases of inflammation of the skin, and as the patient gets better you will find gelatine of ichthyol (10 per cent.) is good. Another application which has a reputation in papular eczema is:—

| | |
|---|---|
| Kaolin ... ... ... ... ... ... | drs. 6 |
| Glycerine ... ... ... ... ... ... | ,, 6 |
| Zinc oxide ... ... ... ... ... | oz. ½ |
| Solution of subacetate of lead ... ... | ,, ½ |

Paint this over the surface and leave it for two hours, and then repaint. In dealing with eczematous ulcers endeavour first to heal the ulceration with an ointment of vaseline, with 2 per cent. of losophan and lysol (1 per cent.). This must be associated with rest in bed. Having got the surface better you will find elastic stockings useful.

*Eczema of the Hands.*—There are certain conditions requiring careful diagnosis when they occur on the palms of the hands—eczema, lichen planus, and psoriasis. Sometimes two of these conditions are combined, and syphilis may complicate any of them. Where fissures occur you will find salicylic-acid plaster very useful; but when the patient has to work, the application of resorcin (3 or 4 per cent.) is useful during the day, and at night the salicylic plaster or ointment. You will also find Lassar's paste useful. Where the eczema involves the fingers, the following has been recommended:—

| | |
|---|---|
| Liquor ferri persulph. ... ... ... | dr. 1 |
| Ung. cerati. galeni. ... ... ... | oz. 1 |
| White wax ... | q.s. to make a consistency. |

In eczema of the nails you are recommended to dip the nails into a solution of 1 drachm of liquor carbonis detergens in about one wineglassful of water, and hold them there five or ten minutes every night.

This exhausts the treatment of eczema, but it is necessary to learn how to apply the various formulæ. The most important thing is to learn when to soothe and when to stimulate. Remember last of all to try to pursue the treatment until you get quite rid of the infiltration, that is until you can pinch up the skin where the eczema has been just as easily as you can the normal skin.

